# Erythropoietin protects lipopolysaccharide-induced renal mesangial cells from autophagy

**DOI:** 10.3892/etm.2014.2124

**Published:** 2014-12-10

**Authors:** LINGYUN BI, RUANLING HOU, DASHENG YANG, SHUJUN LI, DEAN ZHAO

**Affiliations:** 1 Department of Pediatrics, The First Affiliated Hospital of Xinxiang Medical University, Weihui, Henan 453100, P.R. China; 2 Physiology Laboratory, Xinxiang Medical University, Xinxiang, Henan 453000, P.R. China

**Keywords:** erythropoietin, renal mesangial cells, autophagy, p62/sequestosome-1

## Abstract

The aim of this study was to investigate the effects of erythropoietin (EPO) on the impairment of autophagy induced by lipopolysaccharide (LPS) in primary cultured rat glomerular mesangial cells (GMCs). Rat GMCs were isolated and cultured in normal glucose, high-glucose, LPS or LPS + EPO medium. At 24 and 72 h of culture, the cells were examined for expression levels of the autophagy markers LC3 and p62/sequestosome-1 (SQSTM1) using western blot analysis. At 24 h, no significant difference in the expression of LC3 and p62/SQSTM1 was observed among the groups; however, the cells exposed to high-glucose medium for 72 h showed downregulated LC3 expression and upregulated p62/SQSTM1 expression. The cells exposed to LPS (10 ng/ml) for 72 h showed upregulated LC3 expression and upregulated p62/SQSTM1 expression. These changes were reversed in the LPS + EPO group at 72 h. In conclusion, EPO can inhibit LPS-induced autophagy in rat GMCs.

## Introduction

Autophagy exists in higher eukaryotes ranging from yeast to humans. The process is highly conserved and maintains intracellular stability (1). Distinct from the ubiquitin-proteasome pathway, which degrades short-life proteins, autophagy degrades long-life bioplasmin and malfunctional organelles. There a five steps in the autophagy process (2–4): i) Activation of a dual-membrane structure, known as a phagocytic vacuole; ii) amplification of the phagocytic vacuole; iii) maturation of the phagosome; iv) combination between phagosome and lysosome; v) autophagy. In normal physiological conditions, autophagy is maintained at a low level and preserves normal physiological function by cleaning up injured organelles and biomolecules, such as mitochondria and proteins. Dysfunctional autophagy can lead to cancer and degenerative disease; however, whether autophagy participates in the pathogenesis of kidney disease remains unclear. Considerable evidence shows a close association between autophagy and a number of kidney diseases, including acute kidney injury (AKI), diabetic nephropathy and polycystic kidney disease (5). This association could be utilized in the development of novel treatments for kidney diseases.

Erythropoietin (EPO) is a hormone that regulates the generation of erythrocytes. It is primarily produced by the renal cortex and peritubular fibroblasts. The levels of renal, hepatic and cerebral endogenous EPO increase when blood supply or oxygen insufficiency occurs (6). EPO assists oxygen delivery and erythropoiesis and reduces apoptosis, oxidative stress and inflammation (7,8). Several studies have attempted to show the effects of EPO on the kidney, but the mechanism has remained unclear (Jackevicius, Moeini).

The precursor LC3 is divided and formed into LC3-I by autophagy-related gene 4 (Atg4). LC3-I is transformed into the membrane-bound form LC3-II through the activation of Atg7, which is located on the membrane of autophagosomes and autolysosomes (9). LC3-I and LC3-II are indicators of autophagy, and show the activation of autophagy (10). p62/sequestosome-1 (SQSTM1) is an ubiquitin-binding protein, and can induce the autophagy of ubiquitinated proteins by lysosomes in mammalian cells, through cooperation with LC3 (11). The high expression of p62/SQSTM1 can cause an upregulation of the autophagy process and can act as an indicator of autophagy (12).

The aim of the present study was to induce autophagy in rat renal glomerular mesangial cells (GMCs) through the use of lipopolysaccharide (LPS) and to explore the effect of EPO on GMC autophagy to provide novel insight into EPO-mediated renal protection.

## Materials and methods

### Isolation and culture of rat renal GMCs

A total of 32 two-month-old male Sprague Dawley rats were supplied by the Henan Laboratory Animal Center (Zhengzhou, China). This study was carried out in strict accordance with the recommendations in the Guide for the Care and Use of Laboratory Animals of the National Institutes of Health. The animal use protocol was reviewed and approved by the Institutional Animal Care and Use Committee of the First Affiliated Hospital of Xinxiang Medical University (Weihui, China). Both kidneys were isolated from the two-month-old Sprague Dawley rats aseptically. The renal capsule was exfoliated, minced into small pieces and placed on a three-layered stainless steel screen, prior to being washed and ground with a pestle simultaneously. The washing process was terminated when kidney tubules could no longer be observed under the microscope and when >98% of the glomeruli were not associated with the Bowman’s capsule. The glomerular tissue was then collected from the second screen (<75 μm) into a centrifuge tube for centrifugation at 15,000 × g for 5 min. The supernatant was subsequently discarded and the sediment was treated with collagenase V (Sigma, St. Louis, MO, USA) for 10–15 min at 37°C. The reaction was terminated with the addition of RPMI-1640 medium/15% fetal bovine serum (FBS) (Gibco-BRL, Grand Island, NY, USA). The sample was then centrifuged at 15,000 × g for a further 5 min prior to the supernatant being discarded. The glomeruli were collected and seeded in a gelatinized culture flask with RPMI-1640 medium/15% FBS at 37°C and 5% CO_2_. The first passage was conducted after 7–10 days. Twenty-four hours after passage, the cells adhered and formed fusiform or irregular stellate cells. After 3–4 days of culture, the cells formed a tablet.

### Immunofluorescent staining

Immunofluorescent staining was performed by initially activating the cells *in vitro*, staining the surface for antigens and fixing with paraformaldehyde in order to stabilize the cell membrane. The cell membrane was then permeabilized with the detergent saponin in order to allow the antibodies to stain intracellularly. Immunofluorescence showed positive results for monoclonal mouse anti-human α-smooth muscle actin (1:200 dilution; Beyotime Institute of Biotechnology, Shanghai, China) and rabbit anti-human monoclonal vimentin (1:200 dilution; Beyotime Institute of Biotechnology), and negative results for mouse monoclonal cytokeratin (1:200 dilution; Beyotime Institute of Biotechnology) and mouse monoclonal factor VIII (1:200 dilution; Beyotime Institute of Biotechnology). Cells cultured over three passages were used for examination.

### Grouping

The cells were divided into four groups as follows: i) Control (cells were cultured in RPMI-1640 medium/15% FBS and 5 mmol/l glucose; ii) high-glucose (cells were cultured in RPMI-1640 medium/15% FBS and 30 mmol/l glucose; iii) LPS (cells were cultured in RPMI-1640 medium/15% FBS and 10 nmol/ml LPS (Sigma); iv) LPS + EPO (cells were cultured in RPMI-1640 medium/15% FBS, 10 ng/ml LPS and 10 nmol/ml EPO (Sigma). Cells were harvested for examination after 24- and 72-h culture periods.

### Western blot analysis

A total of 0.1 ml pre-chilled radioimmunoprecipitation assay buffer [50 mmol/l Tris-Cl (pH 7.6), 150 mmol/l NaCl, 1% NP-40, 0.1% sodium dodecyl sulfate (SDS), 0.5% deoxycholate, 1 μl/ml leupeptin, 1 μl/ml aprotinin and 0.5 mmol/l phenylmethylsulfonyl fluoride] (Beyotime Institute of Biotechnology) was added to the homogenate and chilled on ice for 30 min. The sample was then centrifuged at 15,000 × g for 30 min, the supernatant was pipetted and the concentration of protein was measured with a bicinchoninic acid protein assay kit (Baiwang Biotechnology Corporation, Shenzhen, China). SDS loading buffer (10 ml) [2X; 2 ml 0.5 mol/l Tris (pH 6.8), 2 ml glycerin, 2 ml 20% SDS, 0.5 ml 0.1% bromophenol blue, 1 ml 1 mol/l dithiothreitol and 2.5 ml ddH_2_O] (TaKaRa, Dalian, China) was added to 30–50 μg protein, and the sample was heated at 100°C for 5 min to denature the protein. Following SDS-PAGE and protein transfer to a nitrocellulose membrane, the membrane was blocked with 1X blocking buffer [5% skimmed milk powder 0.5 g; 20 mM Tris-Cl, (pH 7.5–8.0); 150 mM NaCl; 0.05% Tween-20; Baiwang Biotechnology Corporation] at room temperature for 1 h. The membrane was then immersed in the primary antibody (LC3, dilution ratio 1:2,000; p62, dilution ratio 1:200) (Santa Cruz Biotechnology, Inc., Santa Cruz, CA, USA) and incubated overnight at 4°C. Following incubation with the polyclonal primary antibody (catalogue no. F2426), the membrane was rinsed three times with Tris-buffered saline and Tween^®^ 20 (Baiwang Biotechnology Corporation), with each rinse lasting 5 min. The membrane was then immersed in mouse anti-rabbit secondary antibody (dilution ratio 1:2,000) and incubated at room temperature for 1 h, prior to being rinsed a further three times. A chemiluminescent graph was used to be colored and detected. All of the experiments were performed according to the manufacturer’s instructions (Baiwang Biotechnology Corporation). β-actin was used as an internal control.

### Statistical analysis

All data are expressed as the mean ± standard deviation. Comparisons between two groups were conducted with an independent samples t-test. SPSS 17.0 (SPSS, Inc., Chicago, IL, USA) was utilized for the analysis. P<0.05 was considered to indicate a statistically significant difference.

## Results

**Figure 1:**
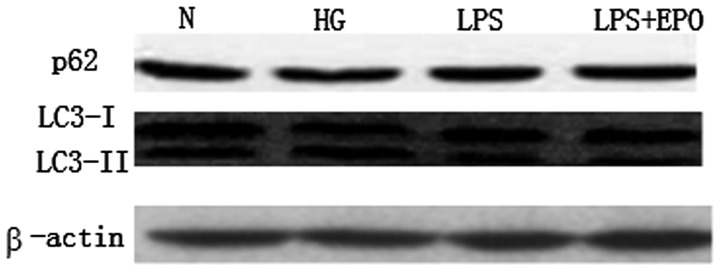
Western blot analysis of LC3 and p62/sequestosome-1 showed no significant differences among the groups after 24 h of culture. N, normal control; HG, high-glucose; LPS, lipopolysaccharide; EPO, erythropoietin.

**Figure 2:**
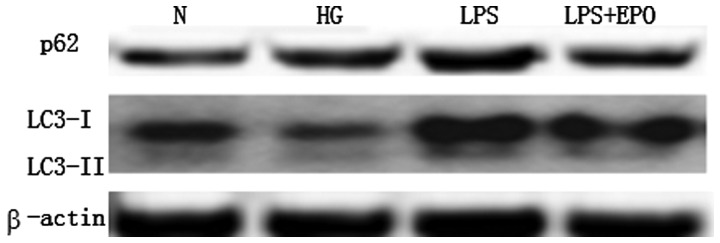
After 72 h of culture, p62/SQSTM1 expression was increased and LC3 expression was decreased in the HG group compared with that in the N group. By contrast, p62/SQSTM1 expression was decreased and LC3 expression was increased in the LPS group. The LPS-induced changes were reversed in the LPS + EPO group. SQSTM1, sequestosome-1; N, normal control; HG, high-glucose; LPS, lipopolysaccharide; EPO, erythropoietin.

In the present study, no significant differences in the protein expression of LC3-I and II, and p62/SQSTM1 were found among the four groups in the 24-h culture experiment (Fig. 1). After 72 h of culture, the expression of p62/SQSTM1 was increased, and that of LC3-I and II was decreased in the high-glucose group compared with the expression in the control group (Fig. 2). By contrast, the expression of p62/SQSTM1 was increased and that of LC3-I and II was increased in the LPS group. The changes were reversed in the LPS + EPO group (Fig. 2). These results suggest that high-glucose conditions can inhibit autophagy in mesangial cells; LPS can induce autophagy in mesangial cells and EPO can suppress this induction.

## Discussion

Several factors, such as diabetes and bacterial infection, can lead to a renal disease, which can seriously affect human health. Autophagy is a common defense mechanism that exists widely in eukaryotes. Autophagy can maintain the intracellular stability by cleaning up damaged or aged organelles and biomolecules, such as mitochondria, peroxisomes, endoplasmic reticulum and paraprotein (13,14). Autophagy therefore plays an important role in pathology. It has been shown that autophagy participates in renal ischemia/reperfusion injury. In a study by Li *et al* (15) it was suggested that high glucose concentrations could inhibit autophagy in mesangial cells, while rapamycin could suppress the high glucose-induced autophagy and N3-methyladenine could upregulate the high glucose-induced autophagy in renal mesangial cells. Renal diseases usually cause anemia, due to an EPO deficiency originating from the renal cortex and renal tubule fibroblasts. As a result, EPO is often administered in renal therapy. In a recent study, Yamaleyeva *et al* (16) treated chronic renal injury with EPO-positive cells and revealed promising results. Furthermore, in a study by Oh *et al* (17) it was found that kidney function could be conditioned using EPO following the occurrence of AKI (17), mainly by improving oxygen transport and erythropoiesis, as well as reducing autophagy, oxidative stress and inflammation (7,8).

In the present study, it was found that high-glucose conditions could suppress autophagy in GMCs; this finding was consistent with the results in the study by Li *et al* (15). The present results suggested that LPS could induce autophagy in GMCs, and that EPO could inhibit the LPS-induced autophagy and protect the renal cells from damage. In an oxidative toxicity rat model, EPO was previously shown to block the autophagy signals in the brain to keep the brain from damage (18). In a recent study by Yu *et al* (19), it was reported that EPO could protect endothelial cells through the same mechanism in a neonatal necrotizing enterocolitis rat model. This indicated that inflammation could induce autophagy and apoptosis and that EPO could block this pathway to protect the cells. The inhibition of autophagy has certain value in clinical practice. A previous study by Zhang *et al* (20) showed that the treatment of folliculin-deficient renal cancer was enhanced through the use of a combination paclitaxel and autophagy inhibitor treatment. Both autophagy inhibition and efforts to target the B-cell lymphoma-2 family of proteins are strategies that could overcome drug tolerance (21). By contrast, Kimura *et al* (22) reported that mitochondrial metabolic stress in renal proximal tubular epithelial cells can lead to a defensive autophagy. Furthermore, the study by Yamahara *et al* (23) showed that obesity-mediated autophagy cannot exacerbate proteinuria-induced tubulointerstitial injury. The role of autophagy in renal disease therefore remains to be fully elucidated.

In conclusion, the present study showed that high-glucose conditions can inhibit autophagy in renal cells, and that LPS can induce autophagy. Furthermore, EPO can reverse this LPS-induced autophagy and protect renal mesangial cells from damage.
